# CytoPacq: a web-interface for simulating multi-dimensional cell imaging

**DOI:** 10.1093/bioinformatics/btz417

**Published:** 2019-05-22

**Authors:** David Wiesner, David Svoboda, Martin Maška, Michal Kozubek

**Affiliations:** Centre for Biomedical Image Analysis, Masaryk University, Brno CZ-60200, Czech Republic

## Abstract

**Motivation:**

Objective assessment of bioimage analysis methods is an essential step towards understanding their robustness and parameter sensitivity, calling for the availability of heterogeneous bioimage datasets accompanied by their reference annotations. Because manual annotations are known to be arduous, highly subjective and barely reproducible, numerous simulators have emerged over past decades, generating synthetic bioimage datasets complemented with inherent reference annotations. However, the installation and configuration of these tools generally constitutes a barrier to their widespread use.

**Results:**

We present a modern, modular web-interface, CytoPacq, to facilitate the generation of synthetic benchmark datasets relevant for multi-dimensional cell imaging. CytoPacq poses a user-friendly graphical interface with contextual tooltips and currently allows a comfortable access to various cell simulation systems of fluorescence microscopy, which have already been recognized and used by the scientific community, in a straightforward and self-contained form.

**Availability and implementation:**

CytoPacq is a publicly available online service running at https://cbia.fi.muni.cz/simulator. More information about it as well as examples of generated bioimage datasets are available directly through the web-interface.

**Supplementary information:**

Supplementary data are available at *Bioinformatics* online.

## 1 Introduction

Ceaseless advances in optical microscopy and fluorescent labelling facilitate *in vivo* studies of cells and their populations. Consequently, as the amounts of acquired bioimage data increase, the need for accurate and robust bioimage analysis raises. This motivates the development of automated image analysis methods to process vast amount of data without the need of manual intervention (Cardona and Tomancak, 2012; Carpenter *et al.*, 2012).

A high demand for automated methods in quantitative microscopy calls for defining objective criteria for their evaluation. To properly evaluate results and performance of bioimage analysis algorithms, suitable benchmark datasets are needed together with their reference annotations (Ljosa *et al.*, 2012). One can obtain such datasets by carefully selecting a reasonable number of naturally varying real bioimages and letting the experts manually annotate them. This is, however, a laborious task with a potential incoherence of results between various specialists (Coutu and Schroeder, 2013), limiting the public availability of such datasets (Kozubek, 2016). To alleviate the existing difficulties, there is an ongoing development of simulators capable of generating synthetic bioimage datasets with accompanying reference annotations as a natural by-product of the underlying simulation processes (Murphy, 2016; Ulman *et al.*, 2016). These simulators often implement complex computational models, allowing *in silico* generation of multi-dimensional bioimage data of a high resemblance to real biological specimens acquired using an optical microscope (Chenouard *et al.*, 2014; Sage *et al.*, 2015; Sorokin *et al.*, 2018; Svoboda and Ulman, 2017).

As the internal complexity of simulation systems naturally increases, working with them becomes progressively more demanding. Setting up these tools generally involves a laborious installation process, often coupled with requirements for a specific operating system and third-party libraries of specific versions. Furthermore, configuration and operation of these tools usually implicate arduous work with a particular programming language or command line interface and a detailed knowledge of complex configuration parameters related to the employed computational models.

In this paper, we present CytoPacq, a modular web-interface for generating synthetic benchmark datasets of various cytology-centric 3D *digital phantoms* (Svoboda *et al.*, 2009), supporting different configurations of the *optical system* and *acquisition devices*, including time-lapse imaging. The web-interface allows users to conveniently configure the whole simulation process by controlling the most important parameters only and by navigating their steps using informative tooltips. Furthermore, advanced options are also available for experienced users, allowing for a detailed configuration of the underlying computational models. The modular architecture built from the ground up with the future-proofing in mind allows the integration of new simulation modules as they become available. The currently implemented simulation modules have already been published and utilized for the generation of bioimage data in multiple on-going, well-established and open-science-supporting research projects.

Datasets, generated using the simulation modules in CytoPacq, have already been used for benchmarking of segmentation and tracking algorithms submitted to the Cell Tracking Challenge (Maška *et al.*, 2014; Ulman *et al.*, 2017), evaluation of image analysis algorithms during development of CellProfiler (McQuin *et al.*, 2018), quantitative assessment of segmentation quality (Stegmaier *et al.*, 2014), validation of image reconstruction via blind deconvolution estimating an unknown point spread function (Keuper *et al.*, 2012) as well as for training of widespread deep-learning approaches (Castilla *et al.*, 2019). Furthermore, the datasets are also included in the well-recognized Broad Bioimage Benchmark Collection (https://data.broadinstitute.org/bbbc).

## 2 Methods and implementation

**Fig. 1.**
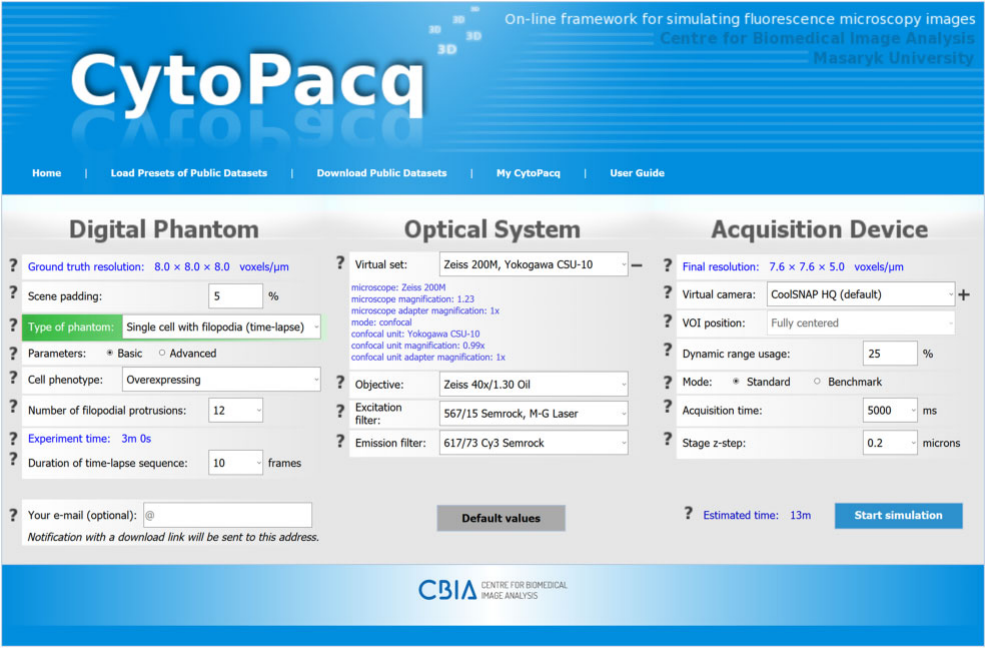
A screenshot of the CytoPacq web-interface. The configuration form is divided into three sections, corresponding to the underlying simulation modules

The CytoPacq web-interface separates configuration parameters into three groups (Fig. 1), depending on the functionality of underlying modules: *3D digital phantom* simulation that generates spatial objects of interest and their structure; *optical system* simulation that models image formation in the optical system; and finally *acquisition device* simulation that mimics the phenomena occurring during image capture when using digital image detectors.

The currently available simulation modules for fluorescently labelled 3D *digital phantoms* are CytoGen (Svoboda *et al.*, 2009), MitoGen (Svoboda and Ulman, 2017) and FiloGen (Sorokin *et al.*, 2018), generating *3D static digital phantoms* of microspheres, HL-60 cell nuclei, granulocyte nuclei and colon tissues, *3D time-lapse digital phantoms* of mitotically dividing HL-60 cells, and *3D time-lapse digital phantoms* of single lung cancer cells with growing and branching filopodial protrusions, respectively. The simulation of a blurring process occurring in the *optical system* is realized in the OptiGen module (Svoboda *et al.*, 2009) via an experimentally measured point spread function. And finally, an image *acquisition* process is mimicked by the AcquiGen module (Svoboda *et al.*, 2009) by including various types of noise and phenomena like sampling and quantization. At present, the web-interface offers a selection of 40 distinct virtual *optical system* configurations and 6 virtual *acquisition devices*, available through the internal database.

CytoPacq is an online service, running all simulations on the dedicated computational servers, thus circumventing the requirement for a powerful workstation on the user side. However, we also endorse the possibility of locally installing the underlying simulation modules on private workstations, thus giving more experienced users complete control over the simulation process, as detailed in the Supplementary Material.

## 3 Concluding remarks

CytoPacq facilitates the generation of theoretically unlimited amount of heterogeneous, completely annotated bioimage data, thus being a valuable resource for objective benchmarking of bioimage analysis methods. Being developed and maintained as a part of the Euro-BioImaging infrastructure (http://www.eurobioimaging.eu) as well as in the frame of COST NEUBIAS networking project (http://neubias.org), its continuous development is well motivated to reflect the needs of the bioimaging community. The modular architecture of CytoPacq opens avenues for integrating new features into it, such as the ability to render multi-channel bioimage data, the support for label-free microscopy modalities, or the possibility of adding new *digital phantoms*.

## Funding

This work was supported by the Czech Science Foundation under the grant No. GA17-05048S (M. M., D. S.), and by the Czech Ministry of Education, Youth and Sports under the grants No. LM2015062 (D. W.), No. CZ.02.1.01/0.0/0.0/16_013/0001775 (M. K.), and No. LTC17016 (D. S.).

*Conflict of Interest*: none declared.

## Supplementary Material

btz417_Supplementary_Data
