# Supplementary material for: CytoPacq: a web-interface for simulating multi-dimensional cell imaging
Source: Bioinformatics. 2019 May 22;35(21):4531–3. doi: 10.1093/bioinformatics/btz417 (PMC6821329; doi:10.1093/bioinformatics/btz417)
Supplement: btz417_Supplementary_Data [file btz417_supplementary_data.pdf]

CytoPacq: A web-interface for simulating multi-dimensional cell imaging

Supplementary Material

User Guide

David Wiesner, David Svoboda, Martin Maška, and Michal Kozubek
Centre for Biomedical Image Analysis, Masaryk University,
Botanická 68a, CZ-60200 Brno, Czech Republic

1. Introduction

CytoPacq is a free web-based service for generating synthetic multi-dimensional images of cells and their populations in optical microscopy with accompanying reference annotations. The aim of the service is to provide an easily-accessible graphical interface for generating benchmark datasets suitable for testing and validation of bioimage analysis methods. Based on the difference from an automatically-generated reference annotation, one can easily compare the quality and accuracy of results of bioimage analysis methods, such as segmentation, tracking, or deconvolution.

Trivia: The web-interface name originates from words "CYTo", "OPTics", and "ACQuisition", where "cyto" originates from Ancient Greek word "kytos", meaning "cell" in english. The connotation of the name itself is "CYTOmetry PACKage".

2. Important Features

- **fully 3D output:** specimens are simulated in arbitrary resolution with configurable z-step
- **time-lapse sequences:** ability to generate dynamic sequences with configurable duration of the experiment
- **phantom fixation:** simulations can be reiterated using previously generated digital phantoms[1]
- **real-life resemblance:** employed computational models cover the most common phenomena occurring during real acquisition process
- **ease of use:** easily accessible web-based graphical interface[2] with contextual help, emphasizing the most important parameters
- **active project:** new modules are incorporated as our development on the synthesis of bioimage data advances

[1] Each generated digital phantom is unique, due to the random nature of the employed computational models, hence the ability to (re)use it as a base for another simulation, with arbitrary configuration of virtual optical system and virtual acquisition device, is crucial for generating benchmark datasets.

[2] Web-interface is working in standard web browsers, i.e. Chrome, Safary, Firefox, Edge, and Opera, with no requirements for additional modules like Java or Flash.

3. Quick Start

After opening the CytoPacq web-interface in your browser, you are presented with the input form divided into three columns, digital phantom, optical system, and acquisition device, corresponding to the underlying modules. The question mark icon on the left side of every parameter row represents a contextual help, explaining the impact of the parameter and offering useful tips. Contextual help for a particular parameter can be accessed by hovering the mouse cursor over the corresponding icon.

You can begin generating data in just two easy steps (see Fig. 1). **First**, you have to select the type of digital phantom that you want to simulate, e.g. a single cell with filopodia. And **second**, you have to click on the “Start simulation” button. Everything else will be handled in the background automatically by the web-interface simulation system.

Note: As the simulation process can take a substantial amount of time, depending on the chosen settings, we do not oblige you to leave the web-interface open in your browser for the whole process. To get back to your simulation later, you can either enter your e-mail (see row marked with an asterisk on Fig. 1) and use the uniquely generated link in the received notification, or you may bookmark the page in your browser.

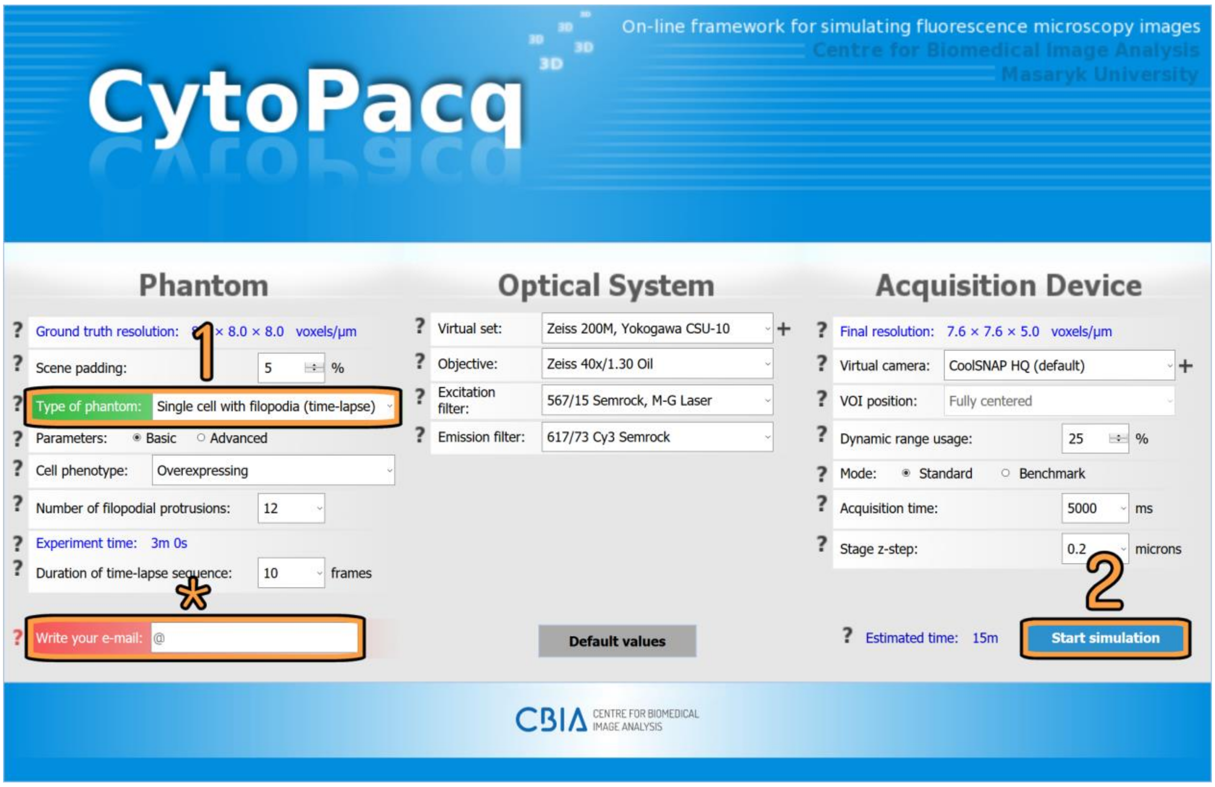

Fig. 1: Configuration form with simulation parameters in the CytoPacq web-interface.

4. Modules

- **DIGITAL PHANTOM**
  The following modules handle the generation of 3D digital cell phantoms (spatial objects imitating cells and their components as well as their structure).
    - **Modeling of static cell populations - CytoGen**
      CytoGen module is capable of generating populations of fixed HL60 cell nuclei, granulocytes, or clusters of human colon tissues.
    - **Modeling of mitosis - MitoGen**
      MitoGen module implements a model of HL60 cell population that evolves in time. All the cells are modelled fully in 3D+time. They can move and split due to mitosis that is also simulated.
    - **Modeling of single cells with filopodial protrusions - FiloGen**
      FiloGen module realizes a model of single lung cancer cells with evolving filopodia. The cell is modeled fully in 3D+time.

- **OPTICAL SYSTEM - OptiGen**
  A module simulating image formation in the optical system, i.e.:
    - blurring process occurring in the optical system
    - uneven illumination
    - virtual excitation/emission filters

- **ACQUISITION DEVICE - AcquiGen**
  A module simulating the phenomena manifesting themselves during image capture using digital image detectors, i.e.:
    - dark current signal
    - (re)sampling
    - fixed pattern noise
    - quantification uncertainty (poisson noise)
    - amplifier (readout noise)
    - A/D conversion (quantization)

5. Workflow

The simulation process (see Fig. 2) begins by specifying desired configuration, which you can easily define by filling the input form in the web-interface (see Fig. 1). This configuration is subsequently passed to the appropriate simulator, depending on the chosen type of digital phantom. The simulation output then consists of synthetic images and accompanying reference annotations.

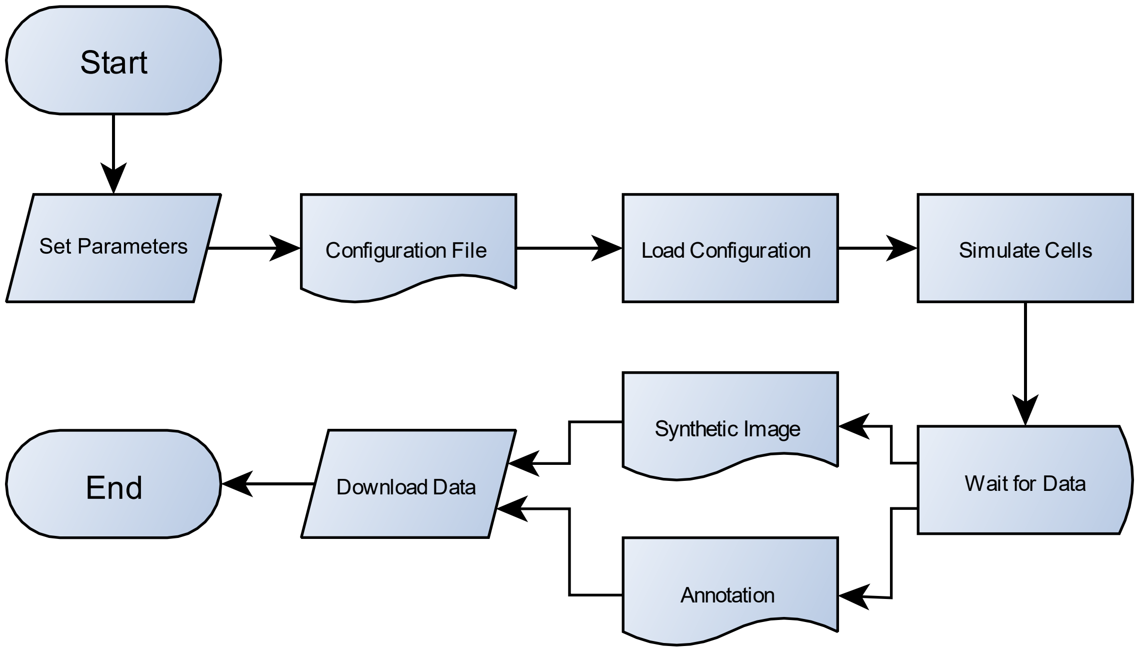

Fig. 2: High-level view (flowchart) of the simulation process.

6. Obtaining the Data

As soon as the simulation is finished, you will be able to obtain the generated data from the download page (see Fig. 3). The download page contains previews of the synthetic images, (selective) downloads of the output data, and links to recommended image viewers.

The image previews correspond to each stage of the simulation process, i.e. digital phantom generation, simulation of optical system, and simulation of acquisition device. You are able to download either complete archive, containing synthetic images of all simulation stages with reference annotations in TIFF or ICS format including generated configuration files, or any of mentioned separately. The recommended viewers, **Viewer 3D** and **Viewer 4D**, are our in-house developed tools for convenient browsing of 3D images and their sequences, respectively.

Note: The generated configuration files in INI or JSON format can be used as an input for our simulators, if you wish to install them on your computer and run them offline. More information about particular simulators can be found at:
https://cbia.fi.muni.cz/research/simulations-and-modeling.html

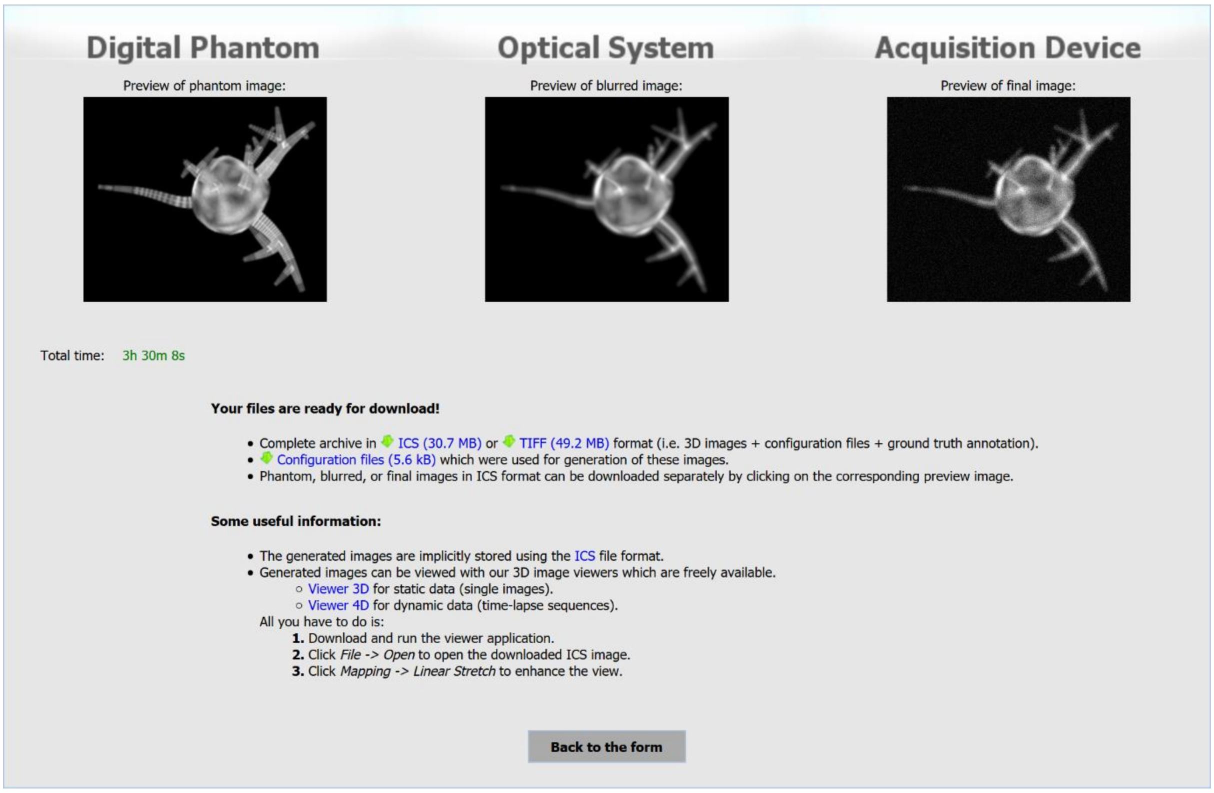

Fig. 3: Download page with a finished simulation.

7. Additional Functionality

The following supplementary services are accessible from the main page.

- **Public Benchmark Datasets and their Preconfigured Parameters**
  Public benchmark datasets, generated by the CytoPacq simulation modules, are available as a part of the Cell Tracking Challenge (CTC, http://celltrackingchallenge.net), Broad Bioimage Benchmark Collection (BBBC, https://data.broadinstitute.org/bbbc), and Masaryk University Cell Image Collection (MUCIC, https://cbia.fi.muni.cz/datasets). Aside from being able to download these public datasets from the main page, user is also offered an option to generate new datasets based on their parameters.

- **My CytoPacq, My Benchmark Datasets**
  My CytoPacq is a personal area, serving as a hub for services facilitating access and manipulation with user data. *My Benchmark Datasets* is a part of *My CytoPacq*, allowing user to access data of their previous simulations at any time directly through the web-interface. Furthermore, the data can be used as a basis for another simulation, i.e. user is able to *fix a phantom* and generate new data using arbitrary configuration of optical system and acquisition device. At the time of writing this text, the phantom fixation is available for microspheres, HL60 nuclei (static and dynamic), granulocyte nucleus, and colon tissue. More services will be added as the development continues. The optional registration is offered to every user who would like to use services in *My CytoPacq*.

8. Running the Software on Own Hardware

As described in Section 3, the simulation has three stages, i.e. simulation of digital phantom, simulation of optical system, and simulation of acquisition device. At the time of writing this text, the CytoPacq web-interface utilizes three simulation modules for digital phantom simulation, CytoGen, OptiGen, and FiloGen. The simulation of optical system and acquisition device is implemented in OptiGen and AcquiGen module, respectively.

All simulation modules are freely available in the form of preconfigured virtual machines, containing the simulation modules and corresponding source codes. All virtual machines can be opened in Oracle VirtualBox (https://www.virtualbox.org). More information about running the software on own hardware can be found on the respective web pages of each simulation module. Essential web links are as follows:

**CytoGen**<br>**OptiGen**<br>**AcquiGen**	Information and virtual machine:	https://cbia.fi.muni.cz/research/simulations/cytogen.html
	License:	GNU GPL
**MitoGen**	Information and virtual machine:	https://cbia.fi.muni.cz/research/simulations/mitogen.html
	License:	GNU GPL
**FiloGen**	Information and virtual machine:	https://cbia.fi.muni.cz/research/simulations/filogen.html
	License:	free of charge for noncommercial and research purposes

9. Terms of use

The usage of datasets generated by the CytoPacq web-interface (or underlying simulation modules) is endorsed for scientific and educational purposes. The usage for any other purpose (e.g. commercial) is generally possible, but solely with the explicit permission of the authors. All generated data is free of charge and may be used as long as the CytoPacq web-interface is properly acknowledged.

More information is available at https://cbia.fi.muni.cz/simulator/terms.php. In case of any questions, please do not hesitate to contact us at cbia-cytopacq@fi.muni.cz.

10. List of Available Optical System Configurations

At the time of writing this text, there are 40 available configurations:

Microscope	Micro-scope adapter	Confocal unit	Confocal unit adapter	Objective	Excitation filter	Emission filter
Zeiss 200M	1x	Yokogawa CSU-10	1x	Zeiss 40x/1.30 Oil	567/15 Semrock, M-G Laser	617/73 Cy3 Semrock
Zeiss 200M	1x	Yokogawa CSU-10	1x	Zeiss 40x/1.30 Oil	650/13 Semrock, M-G Laser	685/40 Cy5 Semrock
Zeiss 200M	1x	Yokogawa CSU-10	1x	Zeiss 100x/1.45 Oil	488/6 Semrock, M-G Laser	528/38 FITC Semrock
Zeiss 200M	1x	Yokogawa CSU-10	1x	Zeiss 100x/1.45 Oil	567/15 Semrock, M-G Laser	617/73 Cy3 Semrock
Zeiss 200M	1x	Yokogawa CSU-10	1x	Zeiss 100x/1.45 Oil	650/13 Semrock, M-G Laser	685/40 Cy5 Semrock
Zeiss 200M	1x	Yokogawa CSU-10	1x	Zeiss 100x/1.45 Oil	488/4, Andor Laser	528/38 FITC Semrock
Zeiss 200M	1x	Yokogawa CSU-10	1x	Zeiss 100x/1.45 Oil	561/6, Andor Laser	617/73 Cy3 Semrock
Zeiss 200M	1x	Yokogawa CSU-10	1x	Zeiss Plan-Apochromat 100x/1.40 Oil	488/4, Andor Laser	528/38 FITC Semrock
Zeiss 200M	1x	Yokogawa CSU-10	1x	Zeiss Plan-Apochromat 100x/1.40 Oil	561/6, Andor Laser	617/73 Cy3 Semrock
Zeiss 200M	1x	Yokogawa CSU-10	1x	Zeiss Plan-Apochromat 100x/1.40 Oil	640/6, Andor Laser	685/40 Cy5 Semrock
Zeiss 200M	1x	Yokogawa CSU-10	2x	Zeiss 40x/1.30 Oil	405/10, Andor Laser	457/50 DAPI Semrock
Zeiss 200M	1x	Yokogawa CSU-10	2x	Zeiss 40x/1.30 Oil	488/4, Andor Laser	528/38 FITC Semrock
Zeiss 200M	1x	Yokogawa CSU-10	2x	Zeiss 40x/1.30 Oil	561/6, Andor Laser	617/73 Cy3 Semrock
Zeiss 200M	1x	Yokogawa CSU-10	2x	Zeiss 40x/1.30 Oil	640/6, Andor Laser	685/40 Cy5 Semrock
Zeiss 200M	1x	Yokogawa CSU-10	2x	Zeiss Plan-Apochromat 100x/1.40 Oil	405/10, Andor Laser	457/50 DAPI Semrock
Zeiss 200M	1x	Yokogawa CSU-10	2x	Zeiss Plan-Apochromat 100x/1.40 Oil	488/4, Andor Laser	528/38 FITC Semrock
Zeiss 200M	1x	Yokogawa CSU-10	2x	Zeiss Plan-Apochromat 100x/1.40 Oil	561/6, Andor Laser	617/73 Cy3 Semrock
Zeiss 200M	1x	Yokogawa CSU-10	2x	Zeiss Plan-Apochromat 100x/1.40 Oil	640/6, Andor Laser	685/40 Cy5 Semrock
Zeiss 200M	0.63x	None	1x	Zeiss 100x/1.45 Oil	365 DAPI Zeiss	445/50 DAPI Zeiss
Zeiss 200M	0.63x	None	1x	Zeiss 100x/1.45 Oil	470/40 EGFP Zeiss	525/50 EGFP Zeiss
Zeiss 200M	0.63x	None	1x	Zeiss 100x/1.45 Oil	550/25 Cy3 Zeiss	605/70 Cy3 Zeiss
Zeiss 200M	0.63x	None	1x	Zeiss 100x/1.45 Oil	640/30 Cy5 Zeiss	690/50 Cy5 Zeiss
Zeiss 200M	0.63x	None	1x	Zeiss Plan-Apochromat 100x/1.40 Oil	365 DAPI Zeiss	445/50 DAPI Zeiss
Zeiss 200M	0.63x	None	1x	Zeiss Plan-Apochromat 100x/1.40 Oil	470/40 EGFP Zeiss	525/50 EGFP Zeiss
Zeiss 200M	0.63x	None	1x	Zeiss Plan-Apochromat 100x/1.40 Oil	550/25 Cy3 Zeiss	605/70 Cy3 Zeiss
Zeiss 200M	0.63x	None	1x	Zeiss Plan-Apochromat 100x/1.40 Oil	640/30 Cy5 Zeiss	690/50 Cy5 Zeiss
Zeiss S100	1x	Atto CARV	1x	Zeiss 63x/1.40 Oil DIC (new)	403/12 DAPI Chroma	457/50 DAPI Chroma
Zeiss S100	1x	Atto CARV	1x	Zeiss 63x/1.40 Oil DIC (new)	490/20 FITC Chroma	528/38 FITC Chroma
Zeiss S100	1x	Atto CARV	1x	Zeiss 63x/1.40 Oil DIC (new)	555/28 Cy3 Chroma	617/73 Cy3 Chroma
Zeiss S100	1x	Atto CARV	1x	Zeiss 63x/1.40 Oil DIC (new)	635/20 Cy5 Chroma	685/40 Cy5 Chroma
Zeiss S100	1x	None	1x	Zeiss 63x/1.40 Oil DIC (new)	403/12 DAPI Chroma	457/50 DAPI Chroma
Zeiss S100	1x	None	1x	Zeiss 63x/1.40 Oil DIC (new)	490/20 FITC Chroma	528/38 FITC Chroma
Zeiss S100	1x	None	1x	Zeiss 63x/1.40 Oil DIC (new)	555/28 Cy3 Chroma	617/73 Cy3 Chroma
Zeiss S100	1x	None	1x	Zeiss 63x/1.40 Oil DIC (new)	635/20 Cy5 Chroma	685/40 Cy5 Chroma
Leica DMRXA	1x	Yokogawa CSU-10	1x	Leica 100x/1.30 Oil	488/10 Chroma, Ar/Kr Laser	525/50 HQ Chroma
Leica DMRXA	1x	Yokogawa CSU-10	1x	Leica 100x/1.30 Oil	568/10 Chroma, Ar/Kr Laser	615/60 HQ Chroma
Leica DMRXA	1x	Yokogawa CSU-10	1x	Leica 100x/1.30 Oil	647/10 Chroma, Ar/Kr Laser	700/75 HQ Chroma
Leica DMRXA	1x	Yokogawa CSU-10	1x	Leica 100x/1.30 Oil	488/4, Andor Laser	525/50 HQ Chroma
Leica DMRXA	1x	Yokogawa CSU-10	1x	Leica 100x/1.30 Oil	561/6, Andor Laser	615/60 HQ Chroma
Leica DMRXA	1x	Yokogawa CSU-10	1x	Leica 100x/1.30 Oil	640/6, Andor Laser	700/75 HQ Chroma

11. List of Available Acquisition Devices

At the time of writing this text, there are 6 available virtual acquisition devices:

- CoolSNAP HQ
- CoolSNAP HQ2
- iXion DV887
- iXion DU888E
- Micromax 1300-YHS
- Quantix KAF-1400

12. Examples of Generated Data

- **HL-60 cell nuclei (fixed cells)**

  Maximum intensity projection of a 3D image in Viewer 3D. Axes XY, YZ, and XZ (left to right, top to bottom).

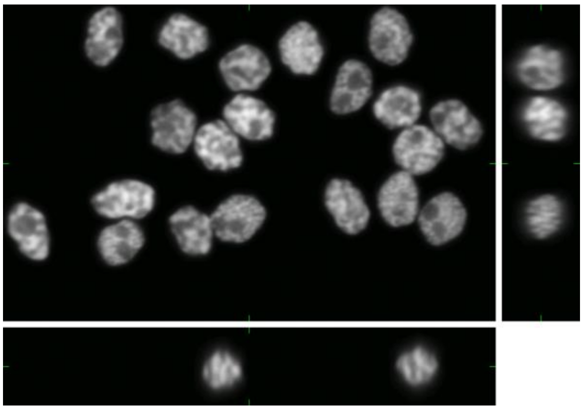

3D image (specimen)

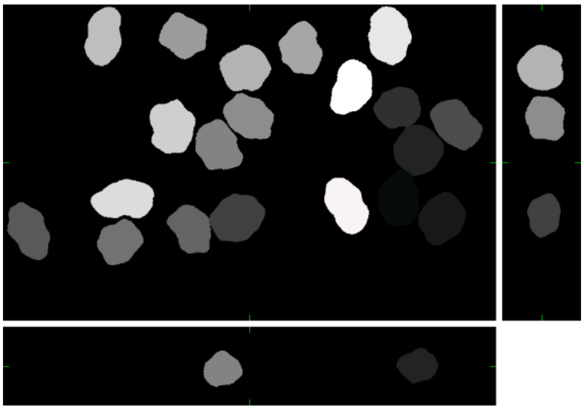

3D labeled image (annotation)

- **Granulocyte nuclei (fixed cells)**

  Maximum intensity projection of a 3D image in Viewer 3D. Axes XY, YZ, and XZ (left to right, top to bottom).

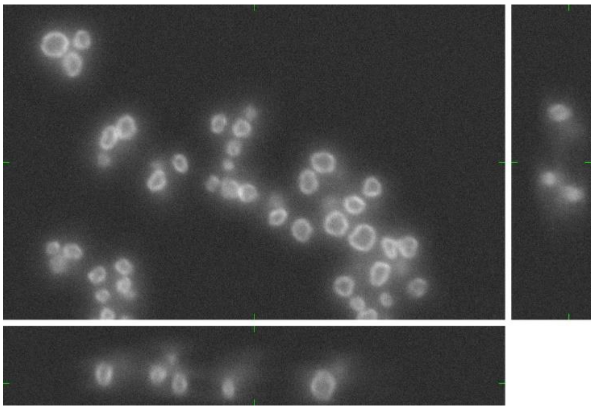

3D image (specimen)

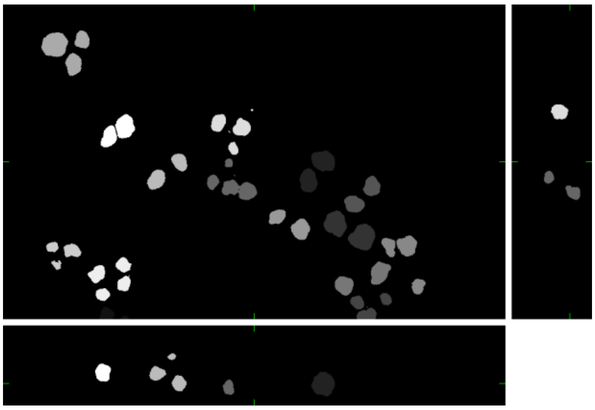

3D labeled image (annotation)

- **Colon tissues (fixed cells)**

  Maximum intensity projection of a 3D image in Viewer 3D. Axes XY, YZ, and XZ (left to right, top to bottom).

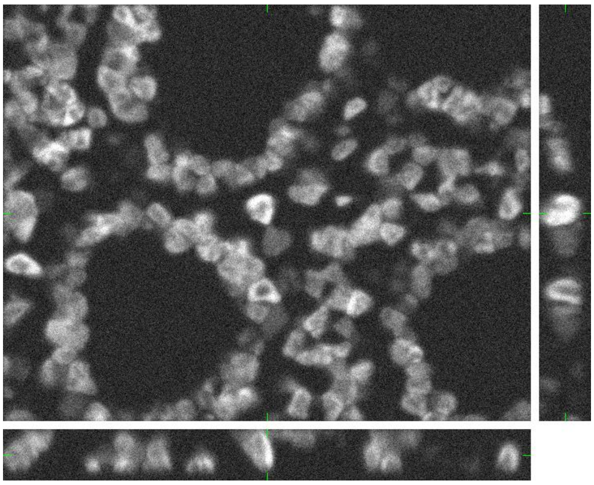

3D image (specimen)

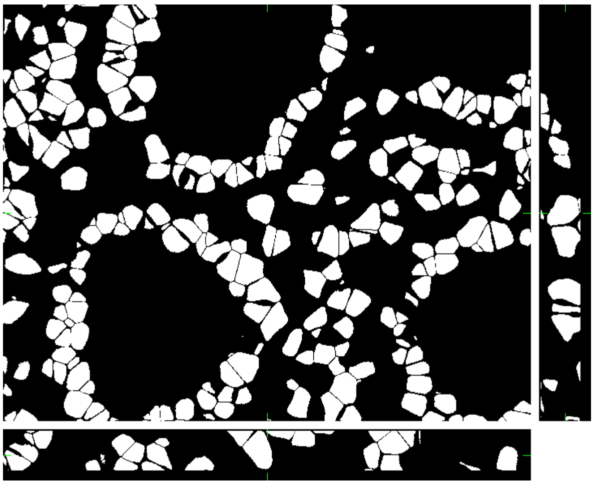

3D mask (annotation)

- **Mitotically dividing HL-60 cells (time-lapse)**

  Rendering of a 3D image sequence in Imaris software.

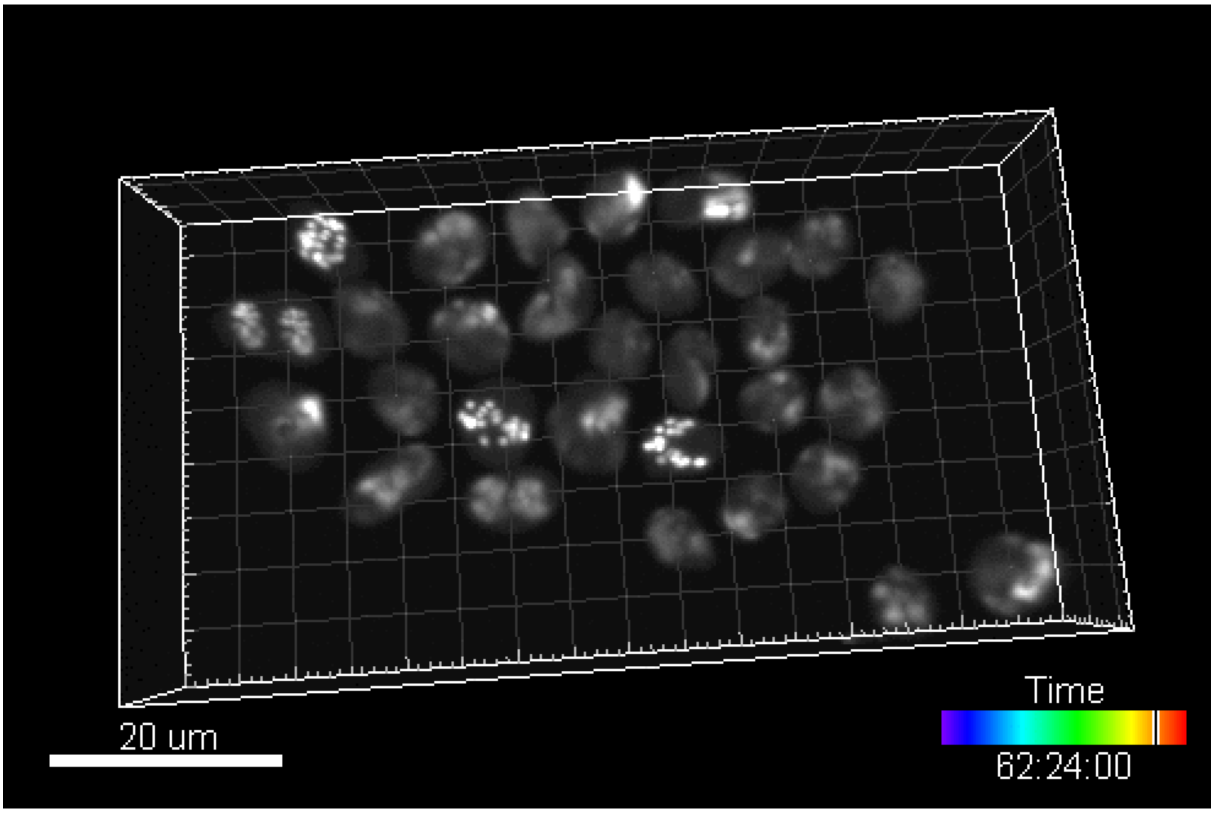

One frame (time-point) of a rendered 3D time-lapse sequence

- **Single lung cancer cell with filopodia (time-lapse)**

  Maximum intensity projection of a 3D image sequence in Viewer 4D. Axes XY and XZ.

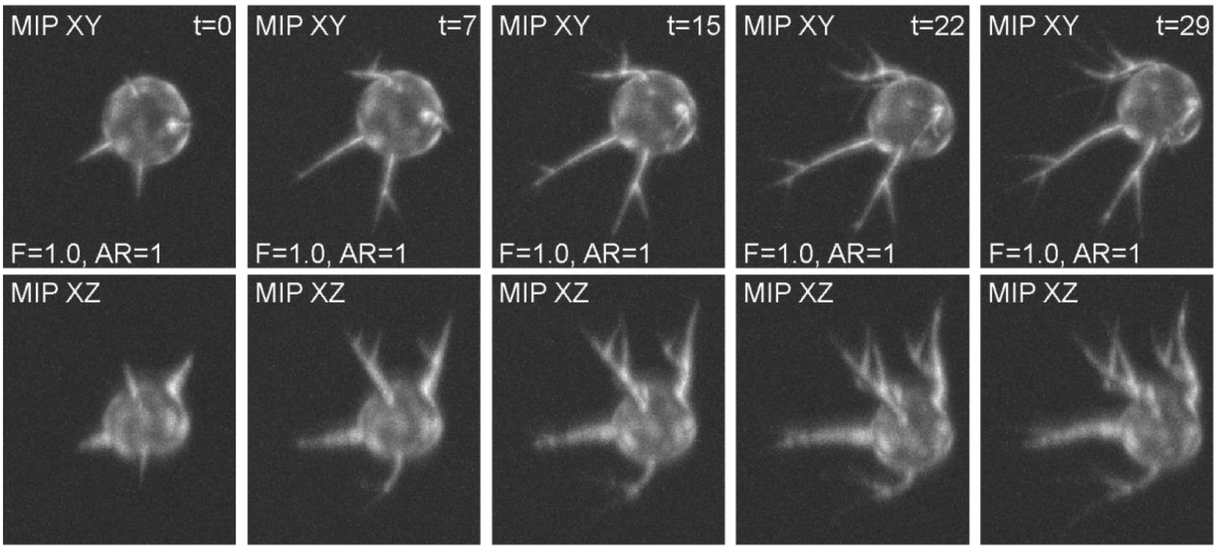

Five frames (time-points) of a 3D time-lapse sequence
